# Annotations

**Published:** 1891-05-09

**Authors:** 


### Annotations.

No man in London has had more abundant opportunities of making himself familiar with every aspect of medical charity than Dr. J. C. Steele, Superintendent of Guy's Hospital. At the meeting of the Royal Statistical Society held a few days ago, Dr. Steele read an exhaustive paper on this perennial question. Two or three weak points which were brought forward by the speaker demand consideration at the present time. One of them is specially urgent. It is this—that the hospitals cannot keep in-patients for so long a period as they ought to keep them, in order that they may be restored to strength as well as health. All hospital workers, who take more than a merely perfunctory interest in their patients, are both grieved and disappointed with the incompleteness which attaches to their work in this respect. Men and women are brought into the wards suffering from illnesses, severe in character, and of considerable duration. In five or six weeks such persons may be out of danger, and may, indeed, be convalescent. If they belonged to the upper or middle classes they would be sent to the seaside, to the country, or even farther a-field for an additional period of several weeks, in order that their strength might be fully brought up to its normal standard. This is the course which ought to be pursued with all convalescents from serious illnesses at hospitals. But it is not done, except in a few of the most severe cases, and that for two reasons; first, because there is such a constant demand made by new patients upon the beds of the hospitals; and secondly, because the hospitals have no sufficient means wherewith to send their patients away. It is not necessary to say that the kind of half cure which the hospitals are thus able to effect is both costly and imperfect, and is as unsatisfactory to the medical charities as to their patients. The sick husband or wife returning to work in a weak and exhausted condition is extremely liable to a second breakdown; and thus the earning power is once more lost, and a second demand is made upon the funds of the hospital. There ought to be available, at least for mothers of families, and all wage earners, a system of convalescent institutions, which would adequately supplement the curative work of the hospitals. We must come to this sooner or later, and we may hope sooner rather than later. If Dr. Steele, or any other member of the Statistical Society, would give us a scheme whereby each hospital patient might not only be cured of his disease, but restored to his normal strength before being sent back to his life struggle, a service of great practical utility to the whole country would be rendered.

Dr. Steele on Medical Charity.

It is startling to learn, on the authority of the chaplain of Milbank prison, that by far the largest proportion of those who fill the ranks of fallen women have originally been domestic servants. Mr. Merrick has taken the trouble to compile a list of memoirs of more than one hundred thousand persons who have passed through Millbank. Of that unhappy number no fewer than fourteen thousand seven hundred and ninety belonged to the fallen women class. It will surprise a good many people to learn that of those fourteen thousand odd rather more than eight thousand had fallen from the ranks of domestic servants, the remaining six or seven thousand being distributed among other female workers in the following proportions: 1,050 were barmaids, 2,267 needle-women, 1,617 trade girls, 228 theatre and music hall employés, 183 governesses, and 167 street-sellers. Thus the domestic servant class contributed more than all the other female occupations put together to the ranks of vice and crime. One would have thought, *à priori*, that an exactly opposite condition of things would have been found, and there would seem to be every justification for such an expectation. Domestic servants are in immediate daily contact with the best educated and most virtuous of women. How, then, does it happen that they, more than any other class of female workers, make shipwreck of virtue? Is it because of their long hours of work, the indifference with which they are treated by their mistresses, and the intolerable dulness of their lives? Most probably it is to some extent. It seems certain that mistresses no longer take the interest in their servants which used to be taken many years ago; it seems certain, too, that in many cases the reason for this is the feeble mental capacity, and the narrow and silly pride and vanity of the mistresses. The modern town-bred woman of the middle class would feel as if she were charged with a monstrous social offence if she were accused of being friendly to her servants. But every woman who employs servants ought to be friendly to them, and ought to make herself as far as possible responsible both for their character and their outward bearing. An intelligent attempt should be made to diminish the dulness of domestic service. Female servants must necessarily work longer hours than most other women, but wherever it is possible they should have relaxations and innocent amusements provided for them, and sufficient intervals of leisure. We are certainly approaching times when much more consideration will be given by all classes to one another than has hitherto been the case. Civilisation means above all things good behaviour, training in conduct, and "sweet reasonableness."

The Dulness of Domestic Service.

Medical science ought to have something to say on the question which is now agitating the minds of the working classes both in our own and in continental countries. No man who takes the trouble to remember the overwhelming number of wage-earners as compared with the rest of the population, and the continued migration of labourers, mechanics, and other workers from villages to large towns, can for a moment doubt the necessity for dealing with questions relating to such a preponderant element of society from every conceivable standpoint which science suggests. It is demanded that the hours of work for large numbers of workmen shall be limited to eight per day. Is it necessary from the point of view of physical or mental health that such limits shall be imposed? This is surely an important aspect of the question; for if it can be shown that the workman has little or nothing to gain in health by such a limitation of the hours of labour, then in the judgment of practical persons a considerable part of his case will have broken down. So far as field labourers and outdoor workers are concerned there is no doubt whatever that work may be continued for twelve hours a day, that is from six in the morning to six in the evening, with proper intervals for meals, without any disadvantage whatever to the workman. After such a day's work, he will still have time enough for rest and recreation in the evening and for abundance of sleep. The average outdoor labourer does not require to sleep more than eight hours. If he discontinues his work at six o'clock in the evening, he may still, after allowing an hour for washing and supper, have two and a-half hours for rest, improvement of the mind, and recreation, before going to bed at half-past nine. The case of indoor workers is somewhat different. They do not have the advantage of working in the open air, and therefore they require outdoor exercise either at midday or in the evening or both. There is no health reason why they should not begin their work at six in the morning and continue it until six in the evening. But inasmuch as they are shut up in close buildings they should have longer intervals for breakfast and dinner than outdoor workers. Moreover, they should have facilities for taking their meals in some other room than that in which they work, and during meal hours the factory and workshop windows should be thrown wide open, fans for setting up air-currents should be at work, and every corner of the work-room should be thoroughly flushed with air at least four times a-day; that is to say, before the commencement of work in the morning, at breakfast time, at dinner time, and after the cessation of work in the evening. If these conditions were fulfilled and facilities were given to factory and workshop hands for securing abundance of exercise in the open air in the evening there is no doubt that a good level of health could be maintained even with a ten or eleven hours' day. This is, as we have said, an exceedingly important aspect of the case. The future of our country is largely the future of the industrial classes. We ought to make it possible for them to live in reasonable comfort and health, and to provide against poverty in old age. If these things can be secured they will be content, and the country may hope to be prosperous. What the working-class needs at the present moment is the "light and leading" of sober, experienced, and disinterested persons. The mere "exploiters" of labour should be shunned, and when they cannot possibly be shunned they should be drowned or hanged.

